# Healthcare professionals’ perceived barriers and facilitators of risk-stratified follow-up care in lung cancer: a qualitative study

**DOI:** 10.1007/s00520-026-10927-0

**Published:** 2026-07-01

**Authors:** Nadia Romana Moss, Nicole Erica Billingy, Irma Maassen, Annemarie Becker-Commissaris, Mireille Broeders, Rosella Hermens, Iris Walraven

**Affiliations:** 1 IQ Health Science Department, Radboud University Medical Center, Kapittelweg 54, 6525 EP Nijmegen, The Netherlands; 2 Department of Pulmonology, Amsterdam University Medical Center, De Boelelaan 1118, 1081 HZ Amsterdam, The Netherlands

**Keywords:** Lung cancer, Follow-up, Risk stratification, Barriers and facilitators

## Abstract

**Purpose:**

Routine follow-up of lung cancer patients involves thoracic computed tomography (CT) scans every 3–6 months for 2 years and then annually up to 5 years. With increasing numbers of survivors, risk-stratified follow-up, tailoring surveillance to recurrence risk, may reduce unnecessary scans while maintaining high-quality follow-up. This study explored healthcare professionals’ (HCPs) perceived barriers and facilitators to implement risk-stratified follow-up in lung cancer care.

**Methods:**

A qualitative study was performed, involving 14 semi-structured individual interviews with HCPs engaged in lung cancer care. Transcripts were analyzed using inductive thematic analysis, with codes subsequently organized according to the Grol and Wensing framework across six different levels: Innovation, Patient, Professional, Social context, Organization, and Economic and Political.

**Results:**

Barriers and facilitators were identified across all levels. HCPs generally viewed risk-stratified follow-up as a promising approach to align care with individual patient risk. Facilitators included its personalized nature, potential to reduce unnecessary imaging, and improve follow-up efficiency. However, HCPs emphasized the need for robust evidence demonstrating safety, effectiveness, and resource optimization. Patient-level barriers included varying follow-up preferences and limited health literacy, while tailored communication was seen as a facilitator. Organizational barriers such as staffing shortages and unclear role delineation were frequently mentioned. Integration into care pathways and interprofessional collaboration were identified as facilitators to address these challenges. Concerns about reduced clinical autonomy and patient safety were also expressed. Financial constraints at the economic and political level were reported to potentially hinder implementation.

**Conclusion:**

HCPs are generally receptive to evidence-based risk-stratified follow-up. Successful implementation requires evidence of effectiveness, integration into care pathways, clear roles, and aligned reimbursement.

**Supplementary Information:**

The online version contains supplementary material available at 10.1007/s00520-026-10927-0.

## Introduction

The introduction of novel therapeutic approaches, such as immunotherapy, has significantly improved lung cancer survival rates [1–3]. Consequently, the simultaneous increase in lung cancer survivors and the growing shortage of healthcare professionals (HCPs) is placing immense pressure on the global healthcare system [4, 5]. Since the current healthcare system is likely not sustainable, novel follow-up strategies are essential to address the needs of lung cancer patients without overwhelming hospital care or merely shifting the workload by transferring cancer survivors to primary care [2].

Routine surveillance is a critical component of follow-up care consisting of three main elements including the type of diagnostic imaging, clinical indication, and follow-up intervals. Currently, in accordance with international guidelines, routine follow-up after curative-intent therapy involves clinical consultations and thoracic computed tomography (CT) scans at least every 6 months for the first 2 years, followed by annual scans for the subsequent three years [6–8]. However, these guidelines are predominantly consensus-based, and there is insufficient evidence for supporting the optimal frequency or modality of follow-up imaging [9, 10]. Consequently, this lack of evidence may lead to both over-surveillance and under-surveillance of lung cancer patients. More evidence-based follow-up care approaches are needed to define appropriate follow-up intervals and imaging strategies that enable timely detection of (oligo)metastatic recurrence, when curative-intent treatment is still feasible.

A potential solution is to offer a more personalized approach, utilizing risk stratification to categorize patients according to their individual risk of lung cancer recurrence or progression [11]. This approach can help to determine the most effective follow-up approach for each risk category, including diagnostic imaging modality and surveillance intervals, thereby reducing the number of unnecessary scans, improving clinical outcomes and optimizing healthcare resource use. Although risk stratification approaches have been proven to enhance lung cancer follow-up care, they have not yet been widely implemented [12–15]. Research shows that HCPs involved in the treatment of various cancer types face barriers to providing risk-based personalized follow-up care, primarily due to inadequate organizational coordination and workforce shortages [16–18]. However, the HCPs’ perspectives on utilizing a risk-stratified follow-up care approach specifically in lung cancer care are currently unknown. Understanding their perspective is essential to inform the design and implementation of a risk-stratified follow-up approach that is feasible, acceptable, and aligned with clinical practice. Therefore, we conducted a qualitative study with the aim to assess HCPs’ perspectives on current lung cancer care and to identify barriers and facilitators for implementing risk-stratified follow-up in lung cancer care.

## Methods

### Study design and setting

We performed a qualitative study, consisting of semi-structured interviews. This study used a thematic analysis approach following Braun and Clarke [19] under an interpretivist epistemological stance. An initial inductive coding process was conducted to identify themes, after which themes relating to barriers and facilitators were organized using the Grol and Wensing framework [20]. The study was conducted according to the consolidated criteria for reporting qualitative research (COREQ) [21]. The study protocol was approved by the research ethics committee METC Oost-Nederland (2023–16243) and compliant with the Declaration of Helsinki. HCPs from eight non-university teaching hospitals and three university teaching hospitals in the Netherlands participated in the study.

### Participants and recruitment

We aimed to include HCPs from various regions across the Netherlands and from different types of hospitals. Thus, a combination of purposive, convenience, and snowball sampling methods was used to recruit HCPs involved in the current follow-up care of lung cancer patients, including pulmonologists, radiation-oncologists, nurse specialists, physician assistants (PA), and lung-oncology nurse consultants. In total, 40 HCPs from 14 different hospitals were approached [22]. Additionally, HCPs affiliated with two local and national lung cancer associations were invited by e-mail or telephone to participate in individual semi-structured interviews. Snowball sampling was employed by asking participating HCPs to refer colleagues who were also involved in lung cancer follow-up care and met the inclusion criteria. All participants had to possess sufficient Dutch language proficiency. Written informed consent was obtained from all participants prior to participation.

### Procedure

Depending on availability, all semi-structured in-depth interviews took place in October and November 2023. Two female researchers (IM and NM) conducted all interviews in Dutch. IM had several years of experience as a research assistant and prior experience in qualitative research methods. NM is a medical doctor and received formal training in qualitative research methods at the start of her doctoral research. Given these backgrounds, the researchers brought prior knowledge of lung cancer care and an interest in implementation-related research. In particular, NM, as a medical doctor with clinical experience in lung cancer care and a potential future interest in specializing in pulmonology, held a positive view towards the implementation of risk-stratified follow-up. This perspective may have influenced the focus on identifying barriers and facilitators, as well as the interpretation of participants’ responses. The second researcher had no direct clinical experience in lung cancer follow-up and approached the topic from a more distanced perspective. The researchers acknowledged that challenges widely reported in literature, alongside those encountered in their own clinical practice, such as workload pressure, organizational, and financial constraints, might be relevant. While not presupposed, and despite efforts to formulate the interview questions as objectively as possible, these considerations may have informed the formulation of the interview guide and the interpretation of participants’ accounts. All participants were aware that the researchers were members of the study team and understood the purpose of the interviews. The multidisciplinary research team engaged in ongoing reflexive practices throughout the study, critically reflecting on how their clinical and research backgrounds, prior experiences, and potential assumptions could have influenced data collection and interpretation. Regular team discussions were used to make these perspectives explicit and support a balanced interpretation integrating the team’s complementary expertise in oncology, epidemiology, and implementation research.

A sample size of 10–15 participants was initially determined based on prior research [23, 24] suggesting that this range may be sufficient to explore key themes. However, data collection was not guided by a predefined saturation threshold. Instead, recruitment and analysis proceeded iteratively. Data were collected and analyzed concurrently, with each interview reviewed to assess the relevance and richness of the data in relation to the research question, as well as the emergence of new themes or codes [23, 24]. Recruitment continued until two consecutive interviews yielded no additional themes. At this point, it was considered to provide sufficient information to address the study aim, and additional interviews were unlikely to contribute substantially to further insights. The interviews were executed in-person, online video conference, or by telephone. Each in-person interview took place on-site at a local (non)university hospital in The Netherlands.

**Fig. 1:**
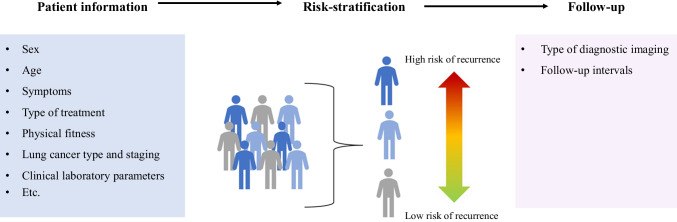
Visual representation of an example of a risk-stratified follow-up care approach

Prior to the interviews, a questionnaire consisting of socio-demographic and occupational details was obtained. All participants received a brief introduction on risk-stratified follow-up care at the beginning of each interview. We gave a hypothetical and illustrative example of risk-stratified follow-up care, using a simplified list of patient-specific factors that could be used to determine a certain risk-category including hypothetical follow-up intervals and diagnostic imaging recommendations for follow-up, shown in Fig. 1. As an illustrative scenario, patients categorized as high risk were described as potentially receiving shorter follow-up intervals, while patients with a low risk might receive extended intervals, with corresponding differences in the type of diagnostic imaging used (e.g., X-ray, CT, or PET-CT). These recommendations were provided for explanatory purposes only. Participants were informed that risk stratification was intended to be dynamic, meaning that risk categories and follow-up recommendations could be reassessed over time based on new clinical information and clinical judgment.

A general topic guide was developed based on the experiences within the research team, literature, and the theoretical framework of Grol and Wensing in order to further standardize the direction of the interviews [20]. This theoretical framework identifies barriers and facilitators at six different levels: Innovation, Patient, Professional, Social context, Organizational, Economic and Political level. The topic guide included questions on HCPs’ perspective on current follow-up care and views on barriers and facilitators at the six distinct levels for implementing a risk-stratified follow-up in lung cancer care. The topic guide translated to English is available in Appendix 1. All interview audio-recordings were transcribed verbatim and anonymized for analysis. The interview transcripts were not returned to participants for verification nor were participants asked to provide feedback on the findings.

### Data analysis

Two researchers (IM and NM) independently coded the transcripts using Atlas.ti Scientific Software Development GmbH version 23.1.1 software. Firstly, all interviews were fully read, and phrases were descriptively labelled by inductive coding [25, 26]. Secondly, codes were reviewed and comparable descriptive codes were grouped into specific subthemes. This process was conducted within a hybrid deductive-inductive approach [25, 26], after which the resulting codes, including both barriers and facilitators, were deductively organized within the Grol and Wensing’s theoretical framework [20] across six levels (Innovation, Patient, Professional, Social context, Organizational, Economic and Political), while the codes themselves were derived inductively from participant data. Consensus meetings with the research group were held to decide on category phrasings, establish a coherent level of interpretation, and to validate preliminary findings. During these meetings, the multidisciplinary perspectives of the research team were considered to strengthen the robustness of the analysis. IBM SPSS Statistics 29 was used to analyze the baseline participants’ characteristics.

## Results

Out of 40 HCPs who were invited via email, a total of 14 HCPs (35%) participated in the semi-structured interviews. After completing these interviews, no new themes emerged, and data saturation was reached. Baseline characteristics of the participants are summarized in Table 1. The majority were female (71%), with a mean age of 54 years. Professional experience ranged from 3 to 30 years (mean = 16 years). Most participants (79%) worked at non-university teaching hospitals.

**Table 1:** Baseline participant characteristics

	Total participants (***n*** = 14)
Age in years, mean (range)	54 (37–64)
Female sex, ***n*** (%)	10 (71)
Work experience in years mean (range)	16 (3–30)
Current profession, ***n*** (%)	
Lung-oncology nurse consultant	2 (14)
Nurse specialist	4 (29)
Physician assistant	1 (7)
Pulmonologist	6 (43)
Radiation oncologist	1 (7)
Healthcare institutions, ***n*** (%)	
University teaching hospital	3 (21)
Non-university teaching hospital	11 (79)

### HCP perspectives on current follow-up care

HCPs described substantial variation in the duration and intervals of follow-up care across institutions, reflecting differences in local protocols. Some HCPs expressed a desire for greater standardization and felt that a clear, nationwide protocol, along with appropriate reimbursement, was lacking. Current reimbursement structures were considered inadequate, particularly for extended consultations or remote follow-ups, which are often not compensated.

I would like to have more time and resources to provide more rehabilitation or physiotherapy and psychosocial options, because fatigue and such are quite an important issue. I really think this would improve follow-up care, because follow-up care is more than CT scans and consultations. (Pulmonologist 1)

High workloads were often mentioned as a barrier to delivering comprehensive supportive care. HCPs reported limited capacity to refer patients to appropriate psychosocial or lifestyle interventions, despite recognizing their importance.

A number of HCPs noted that follow-up care is already somewhat personalized. They emphasized that follow-up care decisions are often based on a combination of patient preference, clinical judgment, disease characteristics, and prognosis. CT scans were reported to be the most commonly used imaging modality, although practices varied: some HCPs alternated between X-rays and CT scans, while others predominantly used CT scans. These choices were generally informed by clinical expertise.

What we do now depends on the doctors’ clinical expertise, choosing to use a CT scan or PET-CT scan, and very occasionally a chest X-ray. (Pulmonologist 2)

Some HCPs mentioned that imaging is sometimes used to mitigate their fear of missing a recurrence and to reassure patients. They mentioned this often led to deviations from current clinical protocols, potentially resulting in excessive use of diagnostic imaging in follow-up care. In general, they encountered lengthy waiting periods for diagnostic imaging opportunities.

Clinical roles in delivering follow-up care also varied by institution. In some hospitals, nurse specialists or PAs independently conducted follow-up visits, which allowed longer consultations and the opportunity to provide supportive care. In other hospitals, pulmonologists retained full responsibility for follow-up care.

I have more time per consultation, so you have different conversations. Of course, I also do my own work in terms of illness and physical complaints and follow-up in terms of scans or laboratory values and such. And then also the regular life around it. (Nurse specialist 1).

### Barriers and facilitators for implementing risk-stratified follow-up care

We found barriers and facilitators for implementing risk-stratified follow-up care on all six levels of the Grol and Wensing framework. An overview of all results and additional quotes can be found in Appendix Tables 1 and 2. Below, we present a detailed exploration of these barriers and facilitators at each level. No meaningful differences were identified, and patterns were consistent across type of HCP or healthcare institutions.

### Level 1: Innovation level

A commonly mentioned prerequisite for the implementation of risk-stratified follow-up care was the need for sufficient evidence demonstrating the effectiveness of risk-stratified follow-up care. HCPs emphasized that clinical judgment must remain a key component in tailoring follow-up care, and that any novel approach should be both evidence-based and practically feasible.

From a HCPs perspective, if follow-up care is individualized per patient and the type of scan or consultation intervals are organized differently, it must remain rational and there must be evidence that it could be done in a different way. (Pulmonologist 2)

HCPs generally believed that risk-stratified follow-up care could optimize resource allocation by reducing unnecessary diagnostic imaging for low-risk patients and enabling more targeted care for high-risk patients.

I think that distributing [resources] based on what someone needs based on their disease burden, allows you to actually provide a little more care for them. And for people who have a reduced health-care burden, you can let go of it a little more. (Nurse consultant 2)

To facilitate implementation of risk-stratified follow-up care, HCPs stressed the importance of user-friendly tools and clear instructions. HCPs agreed that any risk stratification model should include multiple factors, including digital symptom monitoring or patient reported outcome measures (PROMs), physical fitness, health-related quality of life, psycho-emotional wellbeing, social support, patient preferences, and clinical characteristics (e.g., tumor type, tumor markers, smoking behavior, disease trajectory, and vital functions).

### Level 2: Patient level

HCPs generally felt that patients see follow-up care as a source of reassurance. Nonetheless, they anticipated that most patients would accept a risk-stratified care approach, particularly as it offers a more personalized approach. Because you personalize it, it can be preferable in that sense, because you tailor it to the needs of the patient and their requirements. (Nurse consultant 2)

HCPs argued that patients often have varying preferences for follow-up intensity. Some prefer frequent checks, while others seek to minimize hospital visits.

I think you can roughly divide them into two groups. One group wants as many checks as possible and the other wants as few visits to the hospital as possible. (PA)

A key barrier identified was the potential for increased anxiety and uncertainty when follow-up intervals are extended, which may lead to patients refusing to adjust their follow-up intervals.

We always need to be cautious, which makes things tricky-—that grey area, along with the fear and anxiety patients feel at home. Between scans, those uncertain times are common, and the scan provides reassurance. (Radiation oncologist)

In contrast, HCPs noted that for other patients, fewer hospital visits might reduce stress and strengthen their confidence. Fewer hospital visits for some people results in less stress and tension. (Nurse consultant 1)

Health literacy was also identified as a potential barrier to patient engagement of risk-stratified follow-up care. HCPs noted that patients with lower health literacy may require simpler explanations and structured plans, while those with higher health literacy may want a more thorough explanation of the rationale behind follow-up strategies. HCPs emphasized the importance of shared decision-making. Also, encouraging patients to monitor symptoms and to seek care when necessary was seen as especially important for successful implementation.

Patients themselves are their most important controls, if you don’t trust something you can contact us. (Pulmonologist 5)

### Level 3: Professional level

At the professional level, HCPs highlighted a need to balance their own desire for clinical reassurance with the principles of risk stratification. HCPs viewed the risk-stratified model as a supportive tool, not a replacement for their own clinical judgment.

I think it can definitely help if you can say well you really have a low-risk of recurrence so we’re just going to do it this way. Instead of feeling that doubt by yourself (…) when you have a model then you have something to fall back on. (Nurse specialist 4)

HCPs recognized the potential of risk-stratified follow-up care to improve workload distribution, by allocating more time to high-risk patients and reducing unnecessary care for low-risk patients. However, some HCPs argued that extended intervals may reduce direct physician-patient contact moments and the risk of missing clinically significant symptoms between appointments. Consequently, their fear of missing disease recurrence or progression when follow-up intervals are extended was expressed.

Some HCPs also suggested that more frequent follow-up may not always improve follow-up care and that it could increase workload without a clear benefit. A shift in mindset, including clear evidence, more experience, and allowing time to adjust to working with the new approach, was seen as essential for successful adoption.

Especially our own attitudes that we may possibly affect by simply providing evidence, in addition to that there is a need [to change follow-up care] from the patients themselves. (Pulmonologist 1)

### Level 4: Social context

At the social level, several HCPs mentioned resistance to changing established work routines within their clinical practice. Nonetheless, they also felt that improved efficiency and cost-effectiveness might convince colleagues and institutions.

It needs to work well, it needs to be less and eventually save time. It needs to be valid and safe. (Nurse consultant 2)

Moreover, HCPs believed that patients are likely to accept risk-stratified follow-up care if it is clearly explained and embedded within a more patient-centered approach. Shared-decision making, expectation management, and positive framing were considered key facilitators.

If you explain it in such a way that a certain risk category gets more or less, than I think that patients will find it a very good plan as long as patients do get check-ups (Pulmonologist 4)

### Level 5: Organizational level

Organizational constraints, particularly limited imaging capacity and staffing shortage, were identified by HCPs as key barriers. These limitations could especially hinder increased follow-up intensity, as it could result in more consults or scans overall. On the other hand, a decreased follow-up intensity was mentioned to potentially reduce workload and result in less scans.

More frequent is [a barrier] because it requires more scans and time, but when it’s less often it doesn’t matter. (Pulmonologist 1)

Then again if you are low-risk, they won’t need that much care. They can perhaps consult a dietician or something like that in primary care more easily, so that they don’t need to come here [hospital]. (…) It’s just a challenge to set it up right and communicate it well. (Nurse consultant 2)

To support implementation, HCPs emphasized the need for clear and practical communication strategies. There were different views on how best to communicate risk stratification to patients. Some favored personalized advice, while others preferred more general risk categories or quantified risk estimates. Visual tools such as flowcharts were seen as helpful in streamlining implementation. HCPs emphasized the importance of balancing standardization with individualization. While they valued clear risk group recommendations, they also cautioned against overly rigid application.

HCPs often highlighted the potential value of a nurse-led approach to facilitate the implementation of risk-stratified follow-up care. Physicians expressed that they would welcome more involvement of a nurse specialist or PA to administer follow-up care, particularly to support psychosocial and symptom monitoring.

Doctors are actually only involved in technical aspects, and there is much more need for that softer side in follow-up care. Where a nurse specialist can play a valuable role. (Nurse specialist 3)

To embed risk-stratified follow-up care into routine clinical practice, HCPs suggested protocolizing the approach and integrating it into existing clinical care pathways. They argued to incorporate risk-stratified follow-up care in the electronic health record. Here, an adequate information and communication technology (ICT) infrastructure was considered essential. HCPs also highlighted the need for integrated supportive care, especially when follow-up intervals are extended. This includes strengthening collaboration between general practitioners and primary care providers and ensuring that patients have a direct point of contact for questions or concerns.

### Level 6: Economic and political level

At the economic and political level, financial restrictions were seen as an important barrier. Shortened follow-up intervals may raise costs while extending follow-up intervals may result in cost reduction due to fewer scans and visits. However, several HCPs noted that cost savings would only be realized if a larger proportion of patients required fewer scans. This could facilitate better resource allocation and potentially lead to reduced healthcare costs.

If the number of scans can be reduced, which means less burden on the hospital, it will also reduce costs. (PA)

HCPs also pointed to challenges in data sharing, due to the current General Data Protection Regulation (GDPR), which may limit the integration of risk-stratified models in clinical practice. In particular, restrictions on sharing patient information with supportive care providers outside the hospital were seen as a challenge, despite HCPs identifying supportive care as an essential component of risk-stratified follow-up care.

It’s challenging that we cannot access each other’s files, so you’re constantly exchanging a lot of information via fax or email, this is due to privacy [regulations] (Nurse specialist 2)

To support such a recommendation within the context of personalized follow-up care, we may encounter challenges, as data exchange is currently sometimes hindered by the GDPR. (Radiation oncologist)

## Discussion

This study explored Dutch HCPs’ perspectives on the current organization of follow-up care in lung cancer and identified barriers and facilitators for implementing risk-stratified follow-up care. Barriers and facilitators were identified across all six levels of the Grol and Wensing framework [20]. Overall, HCPs viewed risk-stratified follow-up as a promising model to tailor care more precisely to individual patient risk. If supported by strong evidence, such a model was perceived to improve follow-up efficiency, reduce unnecessary imaging, and better align care with individual patient needs.

HCPs emphasized the need for robust evidence demonstrating the clinical safety, effectiveness, and efficiency of risk-stratified follow-up, particularly for reducing unnecessary imaging and workload, through extended follow-up intervals for low-risk patients. A systematic review by Harrison et al. supports these perspectives, emphasizing the need for implementation of evidence-based and innovative care models. It also stated that risk stratification across multiple cancer types may facilitate more efficient allocation of healthcare resources based on individual patient needs, potentially enhancing early detection in high-risk groups while reducing the follow-up burden for those at lower risk [27].

HCPs anticipated that patients would be receptive to risk-stratified approaches, largely due to their personalized nature. They emphasized the importance of adapting communication strategies to health literacy levels to ensure effective patient engagement. Additionally, they highlighted the need to address potential anxiety associated with extended intervals, in particular the fear of recurrence, a concern widely reported among patients with cancer [28, 29]. These findings align with our previous qualitative study exploring patient perspectives patient perspectives on risk-stratified follow-up for lung cancer [30], in which patients likewise viewed risk-stratified follow-up as a promising approach to personalizing care. However, while both patients and HCPs identified proportional use of healthcare resources as an important facilitator and recognized common barriers such as staffing shortages, workflow constraints, privacy regulations, and affordability, their expectations regarding clinical application differed. HCPs emphasized the need for robust evidence of clinical safety and efficiency prior to modifying established follow-up care. In contrast, patients placed greater emphasis on continuity of care and access to a direct point of contact between follow-up consultations, particularly as a means to maintain reassurance when follow-up intervals are extended. Patients highlighted the importance of tailored communication that is responsive to individual concerns. Additionally, both patients and HCPs acknowledged variability in preferences for follow-up intensity, with some patients valuing more frequent follow-up for reassurance and others preferring longer intervals to facilitate a return to normal life. Previous research shows that patient-initiated follow-up and self-management may facilitate personalized care [31, 32] and improve patient satisfaction and perceived quality of care through shared decision-making [33, 34], thereby potentially mitigating unnecessary psychological distress in low-risk patients [35].

The acceptance of risk-stratified follow-up care showed to depend on addressing concerns related to safety, clinical autonomy, and interprofessional collaboration. HCPs viewed risk-stratified follow-up as a tool to support, but not replace, clinical judgment. This perspective aligns with previous research, emphasizing that clinical oversight remains critical in administering risk-stratified follow-up as a decision-support tool [17]. For example, in endometrial cancer, risk stratification can assist HCPs in selecting patients most suitable for risk-stratified follow-up, thereby facilitating care that is responsive to patients’ complex care needs and preferences [17]. Therefore, the successful implementation of such follow-up approaches should be guided by robust evidence and stakeholder input to ensure clinical relevance [36]. Moreover, clear protocols are needed to guide the implementation of risk-stratified follow-up, including a defined mandate under which the approach operates and procedures for resolving discrepancies when its recommendation conflicts with clinical judgment.

Concerns were raised by the HCPs regarding limited imaging capacity and staff shortages, despite optimizing resource allocation by aligning follow-up care with patient risk profiles. These findings align with previous studies identifying that logistical constraints, workforce shortages, and limited resources are common barriers for implementation of innovative healthcare models [16–18, 37]. While most HCPs supported a greater role for nurse specialists. Prior research found that nurse-led models and improved collaboration with primary care are associated with significantly lower healthcare costs compared to specialist-led care [31, 32, 37, 38]. Nonetheless, the broader acceptance of nurse-led models requires cultural change, demonstrable evidence, and a clear delineation of roles.

HCPs further emphasized that demonstrating cost-effectiveness is essential for the implementation and acceptance of risk-stratified follow-up care. They noted that adequate reimbursement systems should be designed for sustainable implementation. Whitin the Dutch healthcare context, current payment models often incentivize the existing “one-size-fits-all” follow-up approach, limiting flexibility for personalized approaches. We acknowledge that these reimbursement-related barriers are context-specific and may not directly translate to other healthcare systems. Although previous research has shown that hospital-based follow-up may not be cost-effective and that personalized and/or nurse-led models could offer similar outcomes at lower costs [31, 32, 37, 38], highlighting the need for aligned reimbursement systems to enable sustainable implementation of innovative risk-stratified follow-up.

A strength of this study is the inclusion of HCPs from diverse occupational backgrounds with varied work experience, and from various (non)university teaching hospitals across the Netherlands, enabling a broad range of perspectives. However, the purposive sampling technique may have introduced participation bias as those with strong views, positive or negative, may have been more likely to participate. Additionally, while the Grol and Wensing framework provided a structured approach for obtaining and analyzing our results, its use may have limited the ability to capture novel themes that could arise from open-coding. Nonetheless, the framework included all topics deemed relevant for implementation of risk-stratified follow-up care, and no new themes were raised by the HCPs during the interviews. Moreover, the patient perspectives from our prior qualitative study [30] are explicitly discussed alongside HCP views, which enables comparison of perspectives across studies. In addition, the sample size was relatively small and included only one radiation oncologist, and the majority of participants were female, which may limit the transferability of the findings to other healthcare settings. Therefore, future studies should include more diverse samples and should evaluate strategies to implement risk-stratified follow-up care.

This study shows that Dutch HCPs are generally willing to adopt risk-stratified follow-up in lung cancer care. Successful implementation requires robust clinical and economic evidence, as well as tools to integrate this model within existing care pathways, and attention to professional and patient-level concerns. Future research should focus on investigating the feasibility, safety, and acceptability of 

## Supplementary Information

Below is the link to the electronic supplementary material.

ESM 1 (DOCX 34.7 KB)

## Data Availability

The data generated and analyzed during the current study are not publicly available due to participant privacy constraints. However, the data may be made available by the corresponding author upon reasonable request and subject to institutional data sharing agreements.
