# Supplementary material for: Healthcare professionals’ perceived barriers and facilitators of risk-stratified follow-up care in lung cancer: a qualitative study
Source: Support Care Cancer. 2026 Jul 1;34(7):713. doi: 10.1007/s00520-026-10927-0 (PMC13323613; doi:10.1007/s00520-026-10927-0)
Supplement: Supplementary file 1 — (DOCX 34.7 KB) [file 520_2026_10927_MOESM1_ESM.docx]

**Supplementary material**

1. TRANSLATED TOPIC GUIDE
2. TABLE 1. BARRIERS AND FACILITATORS PER GROL AND WENSING LEVEL
3. TABLE 2. QUOTES PER GROL AND WENSING LEVEL
4. COREQ CHECKLIST

1. TRANSLATED TOPIC GUIDE

**Introduction**

- Thank the participant for participating in the interview
- Emphasize confidentiality and voluntariness
- Ask permission for audio recording
- Check if the registration form been filled in + informed consent signed
- Check if there are any questions
- Introduce yourself and explain the research goal.
To improve quality of life and follow-up care of lung cancer patients by tailoring this to the individual patient. We want to know what lung cancer patients and care providers involved in follow-up care think about this.

**🡪 Start audio recording including the back-up**

**Perspective on current care***How do healthcare providers experience the current follow-up care approach for lung cancer?*
To start, we are curious about what you think of the current follow-up care approach in your hospital. How would you describe this?

- Ask for clarification, can you tell us more?
- How is the current follow-up care organized? (think of intervals, type of scan, fixed agreements/protocols around it, etc.)
- What is your task in the follow-up care for lung cancer patients?
Have the nurse consultant explain what the difference is with specialized nurse or nurse practitioner (NP).

- What do you think of this current approach?
  - What do you find positive about it?
  - What do you think could be done differently/better and why?

- Do they think that follow-up care should always be done by a specialist?

- What is the ideal follow-up schedule? / What does the ideal follow-up schedule look like?
  - Frequency of consultations: longer or shorter?
  - Type of scan: which type/type of scans do you prefer and why? o Division of roles: who else can provide follow-up care? (Other collaboration, e.g., a larger role for the specialized nurse?)

**Background and brief explanation on risk-stratified follow-up care**

In recent years, the treatment of lung cancer has changed considerably due to new therapies such as immunotherapy. Nevertheless, the follow-up care has remained the same and is primarily expert-based. Lung cancer patients still receive CT scan (or other form of diagnostic imaging) within six months and a check-up with the care provider.

In our study we investigate whether it is possible and desirable to tailor the time between follow-up consultations including the type of scan (CT scan or PET CT scan) to the patient's personal risk of recurrence or progression (metastases, e.g. ) to develop a personalized follow-up care approach. A hypothetical scenario of risk-stratified follow-up care could be that a patient with a high risk of recurrence or progression for example, would receive an advice to come every three months instead of every six months. Whereas low risk patients would have extended follow-up intervals such as eight months instead of six.

- Emphasize that the model is not final and serves as an example!
- When setting up this personalized follow-up care approach we will consider various risk factors including tumor markers and characteristics, lab values, vital functions/ECOG-performance score of a patient, imaging, and symptom course.
- Based on all these patient specific characteristics a personalized follow-up care advice is given for example, high, average, and low risk and also the type of imaging depends on the personalized follow-up care advice.

**Innovation level**I just told you something about personalized follow-up care where the time between consultations and the type of scan are tailored to the personal risk of recurrence or progression.

- When you hear this, what do you think of such an approach? What are your feelings?
- What advantages / disadvantages do you see. Why?
- Do you expect barriers when risk-stratified follow-up care would be applied?
  What could help?
- How should the risk-stratified follow-up care look like?
- How would you like to receive the advice?
  Advice per risk category or individual risk advice?
- Which variables should be included?
- What are the preconditions for using risk-stratified follow-up care? (e.g., knowledge, digital tool in EHR, website, application)

**Patient level**

- What do you think your patients would think of risk-stratified follow-up care?
  - What are advantages or disadvantages?
  - What barriers could they encounter when introducing/using risk-stratified follow-up care.
  - Do you think there are solutions to these barriers?
    If so, how could we achieve this? (do they have sufficient knowledge/skills, attitude, etc.)

**Professional level**

- How do you view risk-stratified follow-up care from your perspective as a HCP?
- What would you think if follow-up care were risk-stratified?
  - Time between appointments shorter or longer
  - Type of diagnostic imaging: CT scan or PET CT scan
- What would you think if the type of imaging changed, for example a PET CT scan instead of CT scan, tailored to the individual risk of recurrence?
- Do you see any advantages to this, if so which ones?
- Do you see any disadvantages to this, if so what kind?
- Do you think there are solutions to these problems?
  If so, how could we achieve this?
- What is needed for you as a healthcare provider to properly implement risk-stratified follow-up care? / What would you need? (e.g., information/knowledge/skills/attitude/(digital)tools)
- Do you think risk-stratified follow-up care could improve care, and if so, how?

**Social context level**Think of our colleagues

- What do you think your colleagues would say about personalized follow-up care?
  - What would be advantages or disadvantages?
  - What would they encounter when introducing/using risk-stratified follow-up care?
  - Do you think there are solutions for these barriers?
    If so, how might we achieve this? (e.g., do they have adequate knowledge/skills, attitude, etc.)

**Organizational level**

- Could risk-stratified follow-up care be implemented in the hospital where you work?
- What barriers do you think they may encounter?(e.g. sufficient staff/capacity, spaces, planning of care/appointments)
- Can these issues be resolved? If yes, how?
- What could help with the introduction of risk-stratified follow-up care?
- Is anything else needed for implementation? (e.g., resources, information, training or (digital) tools)

**Economic and political level**

- What is needed in terms of financing, legislation, and/or policy to make it easier to introduce risk-stratified follow-up care in your hospital?
  Consider: privacy, laws, and reimbursement of care.
- Do you expect any financial problems when introducing risk-stratified follow-up care in your healthcare institution/hospital or department? (e.g., insurance or reimbursement of care)
- Do you expect any legislative problems when introducing risk-stratified follow-up care in your institution? (E.g., social developments or privacy)
- Can the barriers mentioned be overcome?
  If so, how could we achieve this?

**Conclusion: go through questions and see if the participant has any additions.**

**🡪 Stop audio recording and thank participant**

2. TABLE 1. BARRIERS AND FACILITATORS PER GROL AND WENSING LEVEL

| **Table 1a. Barriers** | | | | | |
| --- | --- | --- | --- | --- | --- |
| Innovation | Patient | Professional | Social context | Organizational | Economic and political |
| **Risk categorization challenges:** - Difficulty in classifying lung cancer patients into low/high-risk category - Physicians retain responsibility for medical decisions, including risk assessment of the patient  **Variables** - PROMS may be error-prone | - Patients’ health literacy may influence understanding of different follow-up schemes  - Certain patients may prefer more frequent follow-up visits and resist changes to less intensive schedules.  **Low-risk category** - Patients may experience increased anxiety, insecurity, or fear when follow-up intervals are extended | **Attitude and feelings** - HCP are cautious to rely solely on the output of the risk-stratified follow-up care approach, including the risk estimation and resulting follow-up strategy.  - HCPs have a strong sense of professional responsibility and control  **Low-risk category** - Fear of missing recurrence and/or progression of lung cancer due to extended follow-up intervals - Concern about patient compliance in reporting symptoms - Less frequent physical condition assessment  - Reduced direct patient contact  **High-risk category** - More follow-up care is not always improved care, it may lead to: - Increased workload - More moments of patient contact | **Attitude and feelings** - HCPs are reserved to deviate from their familiar work routines  - HCPs are cautious to accept the risk-stratified follow-up care approach in daily clinical practice | **Hospital logistics and capacity** - Limited radiology capacity and imaging availability  - Staff shortages, especially to provide supportive care  **Professional role delineation** – Some physicians may find it difficult to delegate more follow-up responsibility to nurses or PAs | **Economic** - Insufficient budget to hire additional staff, including specialized nurses  - Standard amount reimbursed for a DBC in follow-up care does not include:  - longer consultation times  - telephone consultations instead of in-person visits  **High-risk category**  - Potentially more costs due to more imaging and/or follow-up consultations - Health insurance companies’ coverages may not cover additional cost due more imaging  **Legislation** - General Data Protection Regulation is too strict, limiting data exchange between institutions involved in risk-stratified follow-up care |

Table 1a The identified barriers per Grol and Wensing level. Abbreviations: Healthcare practitioner (HCP), Patient-Reported Outcome Measurements (PROMS), Positron emission computed tomography (PET-CT) scan, Diagnosis Treatment Combination (DBC), and Physicians assistants (PA).

| **Table 1b. Facilitators** | | | | | |
| --- | --- | --- | --- | --- | --- |
| Innovation | Patient | Professional | Social context | Organizational | Economic and political |
| - May improve follow-up care by reducing unnecessary diagnostic imaging for low-risk patients and enabling targeted care for high-risk patients  **Design risk-stratified follow-up** - Provide sufficient information - Should be easy to use and manageable - Offer either individual or categorical follow-up advice  **Implementation conditions** - Provide scientific evidence supporting risk-stratified follow-up care - Incorporate HCPs’ clinical perspective and assessment - Ensure a direct-point of contact for questions during follow-up  - Risk-stratified follow-up care must be manageable within clinical workflows | **Acceptability**  - Most patients are expected to accept risk-stratified follow-up  - follow-up is generally seen as reassuring and help patients feel more secure about their clinical perspective and prognosis  - Nurses are considered to be more approachable and communicate in a relatable and understandable manner  - Shared decision-making could support implementation of risk-stratified follow-up  **Low-risk category** - For some patients, fewer hospital visits and scans may reduce stress and enhance confidence | **Attitude and feelings** - Most HCPs have a positive view on the risk-stratified follow-up care approach as a supportive tool  - More experience and time to get used to working with the risk-stratified follow-up care approach is necessary to facilitate implementation  - More information and explanation on the use of risk-stratified follow-up care approach are essential  - Potential for improved allocation of workload, allowing more time for high-risk patients  **Low-risk category** - Reduced workload due to fewer consultations - Gradually adjust to less frequent follow-up consultations and develop trust in the risk-stratified follow-up approach  **High-risk** **category**  - Timely detection of recurrence | **Precondition** - The support of HCPs is warranted to adopt the risk-stratified follow-up care approach  **Work environment**  - Ensure clear and consistent communication across HCPs involved in risk-stratified follow-up - Effective collaboration and working arrangement between HCPs in risk-stratified follow-up care are essential  - HCPs are receptive to the argument of cost-effectiveness and resource optimization | - No expected restrictions for implementation  **Work structure and support** - Integrate risk-stratified follow-up into existing care pathways - Provide a clear guideline or protocol on the use of risk-stratified follow-up care approach - Standardize risk-stratified follow-up work practices while allowing individualization - Provide supportive care during risk-stratified follow-up - Incorporate risk-stratified follow-up output in the electronic patient health record  - Ensure sufficient ICT infrastructure and support. - Ensure a direct point of contact during follow-up **Communication** - Foster a good patient-HCP relationship - Provide tailored explanations of risk-stratified follow-up based on patient health literacy - Incorporate shared decision making - Provide comprehensive explanation and information on risk-stratified follow-up care. | **Economic** - Risk-stratified follow-up care should be reimbursed  **Preconditions for implementation risk-stratified follow-up** - Healthcare must remain affordable, with no general increase of costs or medical imaging  **Low-risk** **category**  - Less frequent follow-up may reduce costs due to fewer scans and hospital visits  **Legislation**  - Ensure untroubled sharing of medical data between medical institutions and HCPs involved in risk-stratified follow-up care |

Table 1b The identified facilitators per Grol and Wensing level. Abbreviations: Healthcare practitioner (HCP), Patient-Reported Outcome Measurements (PROMS), Positron emission computed tomography (PET-CT) scan, Eastern Cooperative Oncology Group (ECOG) Performance Status, and Information and Communication Technology (ICT).

1. TABLE 2. QUOTES PER GROL AND WENSING LEVEL

| Table 2. Supportive quotes | | |
| --- | --- | --- |
| **Level** | **Barriers** | **Facilitators** |
| **Innovation** | *“I must say that we still have a few patients who we are very keen to continue with PET-scans. But I have also experienced the disadvantages of PET-scans in the past because you can detect a recurrence very early on that you cannot do anything about or that you will not do anything with because there are no complaints. But then the patient knows that the cancer has returned. Only we are not going to do anything about it. And that is of course very unfortunate. So well, again on indication [perform a PET-scan] and very low-threshold, certainly but not standard for everyone.” (Nurse specialist 4)*  *“I always believed in PET-scans, but the added value has not really been proven. However, you also have different aspects such as the costs and availability. “(Pulmonologist 4 )* | *“Normally, we use a CT-thorax and upper abdomen, but we almost never scan the brain. People can suddenly have 10 brain metastases, and then you really notice doubts in patients. Isn’t something missed? And shouldn’t I have a PET-scan? (Nurse specialist 4)*  *“So, the question is how complete is your follow-up and is it a false sense of security or not? You make all those considerations when you plan that follow-up, but you also want to have a structure. So, if you ask me can it be done differently? I certainly think things can be done differently.” (Pulmonologist3)*  *“I think we can offer quite a bit in terms of follow-up care. And we can make that role somewhat bigger than it’s now. It’s now very much focused on how things are here with my lung cancer, instead of how am I doing as a person”(Nurse consultant 2)* |
| **Patient** | *“Most people are satisfied with a CT scan, but a few insist on a PET-CT scan. The question is, how safe is it to do that often? And how often can you meet those requests?” (Nurse specialist 3)* | *“Open and honest communication with the patient and giving them confidence that these [follow-up consultations] are of course snapshots. And if there is something in between [these snapshot] that they can easily brainstorm with us (…), like what added value does it have to work your way up again with all kinds of unpleasant examinations?” (Nurse consultant 2)* |
| **Professional** | *“There will of course also come a time when we really reach our capacity to determine what we can do in terms of the number of patients that will come our way in the coming decade, which is really is a problem. So, we really will have to make a plan. Here in the hospital, the radiologists have absurd waiting lists because there is a constant flow of scans, and it is also very difficult to stop doctors from that because nothing is as tempting as to do that CT scan. It is also not entirely fair to put that on the patient. Doctors themselves often want to be reassured or know, yes, what is going on.” (Radiation oncologist)* | *“It is mainly expert based: this is how we do it and did it like that for years and this is why. And yes, this is indeed not always entirely appropriate.”(Pulmonologist 6)* |
| **Social context** | *“If you personalize it [follow-up care] you will get more flavors. This can cause confusion among the secretaries and planners or people who conduct the scans. Thus, you really need to state it clearly in your health care status management I think.” (PA)* | *“I can imagine that if you are a patient and you are at low-risk for a recurrence, that it’s nice to hear, of course it gives confidence of the future. Although you probably will not say in so many words that you are at high-risk of recurrence, so we’re going to keep a close eye on you, I think that the positive side can actually help” (Nurse specialist 4)* |
| **Organizational** | *“If it results in more scans, in terms of logistics and availability of the scans can of course be difficult. We already have waiting times, and that will only increase” (PA)* | *“Just put it in the EPD and immediately retrieve your information and provide a proposal.”(Pulmonologist 3)* |
| **Economic and political** | *“The logistics, waiting time and workload are quite important aspects that are already play an important role. And then possibly because of something like that, because of such personalized follow-up care, the intensity of the checks increases, yes that will be difficult. (…) if you want more scans, I think that the complete cost overview and a lot of insurance companies will really make things difficult.” (PA)* | *“One personal [patient] file to which relevant colleagues have access, that would be great.” (Pulmonologist 6)* |

**Table 2** Additional supportive quotes per Grol and Wensing level.

4. Consolidated criteria for reporting qualitative studies (COREQ): 32-item checklist

Developed from:

Tong A, Sainsbury P, Craig J. Consolidated criteria for reporting qualitative research (COREQ): a 32-item checklist for interviews and focus groups. International Journal for Quality in Health Care. 2007. Volume 19, Number 6: pp. 349 – 357

| **Item No** | **Guide Questions/Description** | **Reported on Page #** |
| --- | --- | --- |
| **Domain 1: Research team and reflexivity** | | |
| **Personal Characteristics** | | |
| 1. Interviewer/ facilitator | Which author/s conducted the interview or focus group? | Pg 4, 5 |
| 2. Credentials | What were the researcher’s credentials? E.g., PhD, MD | Pg 1 |
| 3. Occupation | What was their occupation at the time of the study? | Pg 4 |
| 4. Gender | Was the researcher male or female? | Pg 4 |
| 5. Experience and training | What experience or training did the researcher have? | Pg 4 |
| **Relationship with participants** | | |
| 6. Relationship established | Was a relationship established prior to study commencement? | Pg 3, 4 |
| 7. Participant knowledge of the interviewer | What did the participants know about the researcher? e.g. personal goals, reasons for doing the research? | Pg 4, 5 |
| 8. Interviewer characteristics | What characteristics were reported about the interviewer/facilitator? e.g. Bias, assumptions, reasons and interests in the research topic | Pg 4, 5 |
| **Domain 2: study design** | | |
| **Theoretical framework** | | |
| 9. Methodological orientation and Theory | What methodological orientation was stated to underpin the study? e.g. grounded theory, discourse analysis, ethnography, phenomenology, content analysis | Pg 4, 5 |
| **Participant selection** | | |
| 10. Sampling | How were participants selected? e.g., purposive, convenience, consecutive, snowball | Pg 4 |
| 11. Method of approach | How were participants approached? e.g., face-to-face, telephone, mail, email | Pg 4 |
| 12. Sample size | How many participants were in the study? | Pg 5 |
| 13. Non-participation Setting | How many people refused to participate or dropped out? Reasons? | Pg 5 |
| 14. Setting of data collection | Where was the data collected? e.g., home, clinic, workplace | Pg 4 |
| 15. Presence of nonparticipants | Was anyone else present besides the participants and researchers? | N/A |
| 16. Description of sample | What are the important characteristics of the sample? e.g. demographic data, date | Pg 6 |
| **Data collection** | | |
| 17. Interview guide | Were questions, prompts, and guides provided by the authors? Was it pilot tested? | Pg 15, 16 |
| 18. Repeat interviews | Were repeat interviews carried out? If yes, how many? | N/A |
| 19. Audio/visual recording | Did the research use audio or visual recording to collect the data? | Pg 4, 5 |
| 20. Field notes | Were field notes made during and/or after the interview or focus group? | N/A |
| 21. Duration | What was the duration of the interviews or focus group? | N/A |
| 22. Data saturation | Was data saturation discussed? | Pg 4 |
| 23. Transcripts returned | Were transcripts returned to participants for comment and/or correction? | Pg 4 |
| **Domain 3: analysis and findings** | | |
| **Data analysis** | | |
| 24. Number of data coders | How many data coders coded the data? | Pg 5 |
| 25. Description of the coding tree | Did the authors provide a description of the coding tree? | N/A |
| 26. Derivation of themes | Were themes identified in advance or derived from the data? | Pg 5 |
| 27. Software | What software, if applicable, was used to manage the data? | Pg 5 |
| 28. Participant checking | Did participants provide feedback on the findings? | Pg 4 |
| **Reporting** | | |
| 29. Quotations presented | Were participant quotations presented to illustrate the themes/findings? Was each quotation identified? e.g., participant number | Pg 6-10 |
| 30. Data and findings consistent | Was there consistency between the data presented and the findings? | Pg 6-10 |
| 31. Clarity of major themes | Were major themes clearly presented in the findings? | Pg 6-10 |
| 32. Clarity of minor themes | Is there a description of diverse cases or a discussion of minor themes? | Pg 6-10 |
